# SOCIAL AND EMOTIONAL LONELINESS AS PREDICTORS OF COGNITION IN LATER LIFE

**DOI:** 10.1093/geroni/igz038.631

**Published:** 2019-11-08

**Authors:** Bert Hayslip

**Affiliations:** university of north texas, Denton, Texas, United States

## Abstract

To explore the viability of a model illustrating the potential relationship between both social and emotional loneliness and both psychometric and everyday cognition in later life, 575 older adults (M = 73.49) completed measures of crystallized (Gc) and fluid (Gf) ability as well as indicators of self-rated participation in 84 everyday cognitive activities, self-rated stimulatory value of each activity, attitudinal predisposition toward an engaged lifestyle and everyday cognitive failures. Measures of social support, caregiving stress, needs for cognition and cognitive self-efficacy were treated as mediators of the loneliness-psychometric/everyday cognition relationship, controlling for age, gender, health, and education. Hierarchical regression analyses suggested that social loneliness predicted (p < .04) Gc as mediated by social support, cognitive self-efficacy and need for cognition, whereas emotional loneliness similarly predicted (p < .04) Gf. Parallel analyses indicated that social loneliness predicted (p < .04) everyday cognitive failures and that both social and emotional loneliness predicted (p < .03) engaged lifestyle attitudes. In each case, the overall model was statistically significant (p < .01). For everyday cognitive activities and the stimulation values of such, neither type of loneliness was predictive, though lifestyle attitudes and lifestyle activity were moderately interrelated. These findings support a model incorporating distinct dimensions of loneliness as a predictor of diverse aspects of both psychometric and everyday cognition. This suggests that not only are the antecedents of cognition in late adulthood social/emotional in nature, but also that interventions targeting the prevention of loneliness may enhance cognitive functioning in later life.

